# Skin cancers in people with albinism in Togo in 2019: results of two rounds of national mobile skin care clinics

**DOI:** 10.1186/s12885-020-07747-8

**Published:** 2021-01-05

**Authors:** Bayaki Saka, Sefako Abla Akakpo, Julienne Noude Teclessou, Piham Gnossike, Saliou Adam, Garba Mahamadou, Panawé Kassang, Yvette Elegbede, Abas Mouhari-Toure, Tchin Darre, Koussake Kombate, Palokinam Pitché

**Affiliations:** 1 Dermatology and STIs Department, Service de dermatolgie et IST, CHU Sylvanus Olympio, Université de Lomé, BP. 30785 Lomé, Togo; 2 Service de dermatolgie et IST, CHU Campus Université de Lomé, Lomé, Togo; 3 Centre National de Dermatologie de Gbossimé, Lomé, Togo; 4 Service de Chirurgie Maxillo-faciale et Plastique, CHU Sylvanus Olympio, Université de Lomé, Lomé, Togo; 5 Service de dermatolgie et IST, Centre Hospitalier Régional de Tomdè, Kara, Togo; 6 Service de dermatolgie et IST, CHU Kara, Université de Kara, Kara, Togo; 7 Laboratoire d’anatomie et cytotologie pathologique, CHU Sylvanus Olympio, Université de Lomé, Lomé, Togo

**Keywords:** Non-melanoma skin cancers, People with albinism, Togo

## Abstract

**Background:**

In people with albinism (PWA), the deficiency of melanin increase the risk of skin cancers. The aim of this study was to determine the prevalence of skin cancers and characteristics of these detected skin cancers (histological types, localization) in PWA in 10 cities in Togo in 2019.

**Methods:**

This is a cross-sectional study of medical records of PWA systematically examined during two mobile skin care clinics in 2019, as part of a programme for the prevention and management of skin cancers in these subjects.

**Results:**

During the study period, 280 (95.2%) of the 294 PWA consulted, had developed skin lesions. Of the 280 PWA, the pathological reports from the medical records of 33 patients (11.8%; (95%CI = [8.2–16.2]) had concluded to non-melanoma skin cancers. The mean age of these 33 patients was 38.6 ± 15.2 years and the sex-ratio was 1. Their occupations were mainly resellers (21.2%), traders (15.2%) and farmers (12.2%). In the 33 patients, 54 cases of non-melanoma skin cancers were identified, with some patients having more than one tumor, and some of them having more than one (histologically confirmed) diagnosis. These 54 non-melanoma skin cancers were divided into 21 cases of invasive squamous cell carcinomas, 2 cases of Bowen’s disease and 31 cases of basal cell carcinomas. These non-melanoma skin cancers mainly occurred in the head and neck (33 cases; 61.1%), the upper limbs (15 cases; 27, 8%) and the trunk (4 cases; 7.4%).

**Conclusion:**

The results of this study show a high prevalence of skin cancers among PWAs in Togo in 2019, only non-melanoma skin cancers. In addition, they illustrate the role of ultraviolet rays with regard to the localization of skin cancers and the occupations of patients. Popularization and compliance with photo protection measures, systematic and regular examination of the skin of these PWAs will allow early detection and treatment of these skin cancers.

## Background

Melanin, the pigment responsible for skin colour, is photo-protective against carcinogenic solar ultraviolet radiation [1]. Its deficiency in people with albinism (PWA) makes them at-risk to the harmful effects of radiation, namely photophobia, impaired visual acuity and skin cancers. Albinism is a genetic condition affecting approximately 1/17,000 people worldwide [2]. In sub-Saharan Africa, it affects about 1/5000 people [3]. Lack of melanin increases the risk of developing skin cancer a thousand times compared to the general population [2, 4]. Excessive sun exposure is a major environmental factor in accelerated skin ageing, with the development of actinic keratoses and a major risk of skin cancers [2, 5]. These are dominated by non-melanoma skin cancers and are a cause of premature morbidity and mortality of PWA. PWA develop actinic damage at an early age and suffer from skin cancers in the second to fourth decade of life [6, 7]. In most cases, several cancers occur aggressively in those who have not followed protective measures and have had relatively high levels of sun exposure [8]. African PWA are more prone to skin cancers because they live near the equator where exposure to the sun’s ultraviolet rays is very high [9]. In Togo, studies [7, 10] have focused on skin cancers in general, none of which specifically concerned PWA. We felt it necessary to conduct this study to determine the prevalence of skin cancers and characteristics of these detected skin cancers (histological types, localization) in PWA in 10 cities in Togo in 2019.

## Method

Two round of free mobile skin care clinics took place throughout the Togolese territory in 2019 (in 10 cities) in order to treat malignant and premalignant skin lesions in PWA. Togo is located in West Africa with an area of 56,600 km^2^, with a population of 7,352,000 inhabitants as of January 1, 2018 [11]. In Togo, national prevalence is still unknown, but according to previsions [3], the expected total number is about 1470 PWAs. The first round took place in two phases: (i) the first concerned the PWA of Lomé, the capital city, and two cities (Aného, Kpalimé) and was organized in May and June 2019; (ii) and the second phase from 02 to 19 July 2019 covered the cities of Atakpamé, Sotouboua, Sokodé, Kara, Bassar, Mango and Dapaong. The second round also took place in two phases: (i) the first concerned the PWA of the cities of Lomé, Aného, Kpalimé and Atakpamé and was organized from 16 to 30 October 2019; and the second from 10 to 24 November 2019 and covered the cities of Sotouboua, Sokodé, Kara, Bassar, Mango and Dapaong. So, in the 10 cities involved in the dermatological consultation campaigns, the population is estimated at 2,385,535, giving an estimate of 477 PWAs. The mobilization of PWAs in the 10 cities was ensured by the National Association of Albinos of Togo (ANAT) Prefectoral Offices which play the role of sensitizers in the field with the support of the national coordination team. The PWAs were invited through press releases on radio and televison, phone calls, and social networks (WhatsApp). For each campaign, there was the coordination team of the ANAT and the medical team which consisted of a dermatologist, a maxillo-facial surgeon and the ANAT medical officer. Dermatological consultations were provided by a senior dermatologist. All lesions suspected of being malignant or premalignant were removed by surgery (excisional biopsy or simple biopsy) and sent for histological examinations in two facilities in Lomé, by two pathologists. Dermatological consultations were free as well as surgical management of suspicious lesions thanks to the financial support of the Pierre Fabre Foundation and the technical support of togolese dermatological society and the maxillo-facial surgery department. Travel expenses were not covered. However, exceptionally, they had been covered for some PWA who cannot afford to travel to the consultation centers. For this study, we collected data on skin cancers diagnosed in the first campaign, in the second campaign, and even those diagnosed before the two campaigns for some PWAs (by searching in their medical records). For each PWA, the data collected were sociodemographic (age, sex, occupation), clinical and histological (personal and family history, type of carcinoma, localization).

### Ethics approval and consent to participate

This study was approved by the « comité de biothétique pour la recherche en santé » (Ref N° 015/2019/CBRS). We obtained consent from PWAs that participated in the study. For each PWAs, the objectives and benefits of participating in the survey and its conduct were clearly stated, as well as their right to interrupt the interview without justification. An informed consent form signed after the verbal explanation was made by the investigating officer in the language understood by the participant.

## Results

During these two round of mobile clinics, 280 (95.2%) of the 294 PWA examined had presented skin lesions. Among them, 115 biopsies/excisional biopsies were carried out for histological examination in 79 patients, some patients had more than one biopsy/excisional biopsy. Of 115 biopsies/excisional biopsies, 54 (46.9%) returned as malignant in 33 (41.8%) of the 79 patients who underwent skin biopsy/excisional biopsy; some patients having more than one tumor, and some of them having more than one (histologically confirmed) diagnosis. So, of the 280 PWA who had presented skin lesions, pathology reports from the records of 33 patients (11.8%; (95%CI = [8.2–16.2]) had concluded that skin cancer, only non-melanoma skin cancers, were present. The mean age of these 33 patients (17 males and 16 females) was 38.6 ± 15.2 years (extremes: 15 and 75 years). The median age was 37.5 years [range: 28–46 years], the median age of patients with squamous cell carcinoma (SCC) and basal cell carcinoma (BCC) was 39 years [range: 28–46 years] and 35.5 years [range: 28.5–46.5 years], respectively. Their occupations were predominantly resellers (21.2%), traders (15.2%) and farmers (12.2%) (Table 1).

**Table 1:** Occupation of Persons with Albinism with skin carcinomas

	Number	%
Reseller	7	21,2
Retailer	5	15,2
Farmer	4	12,2
Student	4	12,2
Housewife	4	12,2
Topographer	1	3,0
Taxi driver	1	3,0
Computer scientist	1	3,0
Unemployed	1	3,0
Pastor	1	3,0
Teacher	1	3,0
Infographer	1	3,0
Management Assistant	1	3,0
Mechanic	1	3,0
**Total**	**33**	**100,0**

Seventeen of the 33 patients had a single non-melanoma skin cancers, compared to 16 patients who had several cases or types of non-melanoma skin cancers (11 had two and 5 had three). With respect to histological type, the 54 non-melanoma skin cancers were divided into 21 cases of invasive SCC (38.9%), two cases of Bowen’s disease (3.7%) and 31 cases of BCC (57.4%). Of the 16 patients with more than one non-melanoma skin cancers, six patients had only BCC, three had only SCC, and seven had both BCC and SCC (Table 2). These non-melanoma skin cancers were predominantly in the head and neck (33 cases; 61.1%) (Table 3).

**Table 2:** Skin carcinomas in persons with albinism

	Number	%
**Number of patients with 1 single skin carcinoma**	**17**	**51,5**
*Number of patients with 1 SCC*	*8*	*27,3*
*Number of patients with 1 BCC*	*9*	*24,2*
**Number of patients with 2 skin carcinomas**	**11**	**33,3**
*Number of patients with 2 BCC*	*5*	*15,1*
*Number of patients with 2 SCC*	*2*	*6,1*
*Number of patients with 2 Bowen’s disease*	*1*	*3,0*
*Number of patients with 1 SCC and 1 BCC*	*3*	*9,1*
**Number of patients with 3 skin carcinomas**	**5**	**15,2**
*Number of patients with 2 BCC and 1 SCC*	*2*	*6,1*
*Number of patients with 1BCC and 2 SCC*	*2*	*6,1*
*Number of patients with 3 BCC*	*1*	*3,0*
**Total (54 carcinomes cutanés)**	**33**	**100,0**

*BCC* Basal cell carcinoma, *SCC* Squamous cell carcinoma

**Table 3:** Site of Skin Carcinomas

	SC, number (%)	SCC, number (%)	BCC, number (%)
Head and neck	33 (61,1)	17 (74,0)^a^	16 (51,6)
Upper limbs	15 (27,8)	4 (17,4)	11 (35,5)
Trunk	4 (7,4)	1 (4,3)	3 (9,7)
Lower limbs	2 (3,7)	1 (4,3)	1 (3,2)
**Total**	**54 (100)**	**23 (100)**	**31 (100)**

*BCC* basal cell carcinoma, *SCC* Squamous cell carcinoma, *SC* skin carcinomas, ^a^including the two cases of Bowen’s disease

## Discussion

In our study, the prevalence of non-melanoma skin cancers was 11.8%, lower than the 26% reported in Brazil [8], 25% in Tanzania [12], 23% in South Africa [13] and 20,98% in Nigeria [14], but higher than the 4.6% reported in France [15]. While the mobile consultation strategy is the one we have used in the campaigns, the majority of our consultations are limited to urban PWAs, because other PWAs in more remote areas, which do not even have access to health care centres, have not been affected by these activities. The low prevalence in our study can be explain by the fact that PWAs without skin cancer seemed to have been more likely to participate in this campaigns. Indeed, the risks of skin cancers is lower in urban PWAs beacause they have more access to awareness-raising actions on the media on photo protection than rural PWAs, and protect themselves better. Moreover, PWA are the target of prejudice and social exclusion and have limited access to specialized medical care and resources [8, 16].

The average age of patients in this study was 38.6 years, much lower than that of the general Togolese population (42 years) [10]. However, it is similar to the 35.5 years found in Tanzania [6] with an earlier onset of BCC (median age 35.5 years) compared to SCC (median age 39 years). PWA develop sun damage earlier due to lack of sun protection (covering clothes, wide-brimmed hats, sunscreen, indoor occupations to avoid repeated sun exposure), so malignant tumours occur from the second decade of life [2, 6].

We have identified 54 cases of skin carcinomas dominated by BCC (57.4%), with some patients having more than one tumor, and some of them having more than one (histologically confirmed) diagnosis. This finding of multiple lesions has already been made in the literature in this population [8, 9, 17, 18], including 14% (4 cases) of combined BCC and SCC in Brazil [8], one case of multiple BCC in India [17] and one case of SCC, Bowen’s disease in Japan [18]. In our study, there was a predominance of BCC with a BCC/SCC ratio of 1.47. In Nigeria [14], malignant skin lesions comprising 55% of BCCs, 22% of SCCs, 18% basosquamous carcinoma and 5% collision tumour (BCC and SCC). In Brazil [8], it was 62% BCC, 51% SCC and 7% melanoma. These three studies including ours, are cross sectional community based studies. However, most African studies, all retrospective hospital based, show that SCC is the most common cancer in this population [4, 6, 9, 19]. In Tanzania, there were 72 cases (53.7%) of SCC versus 61 (45.5%) of BCC and one case of melanoma (0.75%) [6]. In another study in Nigeria, there were 68.2% SCC, 22.7% BCC and 9.1% melanoma [9]. A review had shown that SCC was observed in 5–88% of cases compared to 9–23% and 1.3–3% respectively for BCC and melanoma [20]. Indeed, the incidence of SCC doubles with every 8–10 degrees of decline in latitude with a maximum incidence at the equator [6]. Finally, melanoma is rare in PWA with a similar incidence in the general population [8], a tumour that we did not find in our study.

Skin cancers develop at sites in the body exposed to ultraviolet radiation [4, 19, 21]. In our study, the lesions were mainly located in the head and neck (33 cases; 61.1%), followed by the upper limbs (15 cases; 27.8%). Head and neck are similar locations reported in other studies [14, 21, 22]. Resellers (21.2%), traders (15.2%) and farmers (12.2%) were the predominant occupations in our study. In Nigeria [9], these were artisans and farmers. These activities chronically expose the head and neck to the sunlight, which is the major risk factor for skin cancer [5, 21].

### Limitations of study

One of the limitations of this study is that the two campaigns concerned only 10 towns in Togo, without forgetting that rural areas were not concerned. Although awareness campaigns and statements have been advertised to invite PWA to these campaigns, the number of PWA reached by these activities is far below the estimates of the number of PWAs in Togo according to the National Association of Albinos in Togo (ANAT). Those who lived further away may not have participated either because not knowing of the initiative or because being a day away from work in addition to travel costs would be too expensive. Secondly, the low prevalence of non-melanoma skin cancers in our study can be explain by the fact that PWAs without skin cancer seemed to have been more likely to participate. Indeed, the risk of skin cancers is lower in urban area because of awareness-raising actions on the media on photo protection.

## Conclusion

The results of this study showed a high prevalence of skin cancers in PWA in Togo in 2019, only skin carcinomas dominated by basal cell carcinomas. Moreover, their location in photo exposed areas, and the occupation being photo exposed illustrate the role of ultraviolet radiation. The popularization and respect of photo-protection measures, systematic and regular examination of the skin of these PWA will allow early detection and management of these skin cancers.

## Data Availability

Extracted data are with the corresponding author and available under reasonable request.

## Abbreviations

ANAT: National Association of Albinos
BCC: Basal cell carcinoma
NGO: Non-Governmental Organization
PWA: Patients with albinism
SCC: Squamous cell carcinoma
SOTODERM: Togolese Society of Dermatology and Sexually Transmitted Infections
UV: Ultraviolet
